# Forecasting the effects of vaccination on the COVID-19 pandemic in Malaysia using SEIRV compartmental models

**DOI:** 10.4178/epih.e2023093

**Published:** 2023-10-17

**Authors:** Mei Cheng Lim, Sarbhan Singh, Chee Herng Lai, Balvinder Singh Gill, Mohd Kamarulariffin Kamarudin, Ahmed Syahmi Syafiq Md Zamri, Cia Vei Tan, Asrul Anuar Zulkifli, Mohamad Nadzmi Md Nadzri, Nur'ain Mohd Ghazali, Sumarni Mohd Ghazali, Nuur Hafizah Md Iderus, Nur Ar Rabiah Binti Ahmad, Jeyanthi Suppiah, Kok Keng Tee, Tahir Aris, Lonny Chen Rong Qi Ahmad

**Affiliations:** 1 Institute for Medical Research (IMR), National Institutes of Health (NIH), Ministry of Health Malaysia, Setia Alam, Malaysia; 2 Department of Medical Microbiology, Faculty of Medicine, University of Malaya, Kuala Lumpur, Malaysia

**Keywords:** COVID-19, Vaccination, Epidemiological models, Malaysia

## Abstract

**OBJECTIVES:**

This study aimed to develop susceptible-exposed-infectious-recovered-vaccinated (SEIRV) models to examine the effects of vaccination on coronavirus disease 2019 (COVID-19) case trends in Malaysia during Phase 3 of the National COVID-19 Immunization Program amidst the Delta outbreak.

**METHODS:**

SEIRV models were developed and validated using COVID-19 case and vaccination data from the Ministry of Health, Malaysia, from June 21, 2021 to July 21, 2021 to generate forecasts of COVID-19 cases from July 22, 2021 to December 31, 2021. Three scenarios were examined to measure the effects of vaccination on COVID-19 case trends. Scenarios 1 and 2 represented the trends taking into account the earliest and latest possible times of achieving full vaccination for 80% of the adult population by October 31, 2021 and December 31, 2021, respectively. Scenario 3 described a scenario without vaccination for comparison.

**RESULTS:**

In scenario 1, forecasted cases peaked on August 28, 2021, which was close to the peak of observed cases on August 26, 2021. The observed peak was 20.27% higher than in scenario 1 and 10.37% lower than in scenario 2. The cumulative observed cases from July 22, 2021 to December 31, 2021 were 13.29% higher than in scenario 1 and 55.19% lower than in scenario 2. The daily COVID-19 case trends closely mirrored the forecast of COVID-19 cases in scenario 1 (best-case scenario).

**CONCLUSIONS:**

Our study demonstrated that COVID-19 vaccination reduced COVID-19 case trends during the Delta outbreak. The compartmental models developed assisted in the management and control of the COVID-19 pandemic in Malaysia.

## GRAPHICAL ABSTRACT

[Fig f6-epih-45-e2023093]

## INTRODUCTION

The coronavirus disease 2019 (COVID-19) emerged in late December 2019 and the World Health Organization (WHO) declared the end of the global health emergency on May 5, 2023. During this period, the disease resulted in 768 million cases and 6.94 million deaths worldwide [1,2]. In Malaysia, there have been more than 5 million COVID-19 cases and 37,000 fatalities to date [3].

The COVID-19 pandemic has had a profound impact on health, societies, and economies across the globe. Countries worldwide have implemented strategies such as public health social measures (PHSMs) and vaccination to combat the pandemic. To evaluate the effectiveness and impact of PHSMs and vaccination on the course of COVID-19 transmission, disease forecasting using deterministic compartmental models like susceptible-infectious-recovered and susceptible-exposed-infectious-recovered models has been widely employed during the pandemic [4,5]. These models have incorporated simulated scenarios of varying vaccination coverages, rates, efficacies, vaccine allocation strategies, and strategies to ease PHSMs during the vaccination roll-out. They have been instrumental in guiding public health interventions and policy-making [5-13]. For instance, a study conducted in China by Zhao et al. [6] concluded that it would be necessary to vaccinate at least 85% of China’s total population with a vaccine having an efficacy of over 70% to halt COVID-19 transmission before considering lifting PHSMs. It has been demonstrated that increasing vaccination rates and coverage can reduce the number of new COVID-19 cases and hospitalizations, as reported in the United States and China [6,7]. However, in the context of limited vaccine supply, particularly in low-income and middle-income countries, the optimal strategy to mitigate complications from COVID-19 and disease transmission has been to prioritize vaccine allocation for the older population first [6,10].

In Malaysia, the National COVID-19 Immunization Program (NIP) was initiated on February 24, 2021. This involved the procurement of 44,799,300 doses of the Pfizer vaccine, 12,400,000 doses of the Sinovac vaccine, and 6,400,000 doses of the AstraZeneca vaccine for the year 2021 [14,15]. The NIP was carried out in 3 phases. The first phase, which began in February 2021, aimed to vaccinate approximately 500,000 essential service workers to ensure the continuation of healthcare delivery and essential public sector services during the pandemic [15]. The Pfizer vaccine was administered during this phase [16]. The second phase commenced in April 2021, with the goal of vaccinating 9.40 million individuals aged 60 and above, as well as people with chronic illnesses [15]. The third phase aimed to vaccinate the remaining 13.70 million adults aged 18 and above, starting from June 21, 2021, in an effort to control the spread of the disease [15,17]. The Pfizer, AstraZeneca, and Sinovac vaccines were administered during the second and third phases [16]. The initial objective of the NIP in Malaysia was to have 80% of the adult population fully vaccinated (having received 2 doses of the vaccine) by February 2022 [15]. However, during the third phase of the NIP, the highly transmissible Delta variant of concern (VOC) began to circulate, leading to the largest outbreak of the year 2021 [18,19]. Over 2 million COVID-19 cases and 27,000 COVID-19 deaths were recorded during this phase, accounting for nearly 78% of all COVID-19 cases and 87% of all COVID-19 deaths in 2021 [20]. In response to the increased virulence of COVID-19 and the rapid transmission of the Delta VOC, a revised goal was proposed: to have 80% of the adult population fully vaccinated between October 31, 2021 and December 31, 2021 [21].

Numerous studies have utilized compartmental models to investigate the impact of vaccination on the COVID-19 pandemic [5,7-9,12]. However, in Malaysia, there have been few studies that simultaneously consider the proportion of vaccine allocation, vaccine efficacy, and vaccination rate. Therefore, this study aimed to analyze the effects of COVID-19 vaccination on daily case trends during Phase 3 of the NIP in Malaysia. We employed susceptible-exposed-infectious-recovered-vaccinated (SEIRV) compartmental models that concurrently accounted for the efficacy of the vaccine against the Delta VOC, the proportion of vaccine allocation, and the vaccination rate. The objective was to achieve full vaccination in 80% of the adult population by both October 31, 2021 and December 31, 2021.

## MATERIALS AND METHODS

### Data source

The daily COVID-19 case data from June 21, 2021 (the beginning of Phase 3 NIP) through December 31, 2021, as well as the daily COVID-19 vaccination data from June 21, 2021 to July 21, 2021, were obtained from the official online repository of Malaysia’s Ministry of Health (MOH) [20]. Information regarding the procurement of Pfizer, Sinovac, and AstraZeneca vaccine doses was gathered from the 2021 report of the Public Accounts Committee of the Fourteenth Parliament [14]. Vaccine efficacy data was derived from published literature that offered information on the levels of vaccine efficacy against the Delta variant [22-24].

### Model development

The SEIRV model was constructed using the ODIN interface of the R programming software, a tool developed by Imperial College London [25]. Initially, the SEIRV model was fitted using parameters derived from local authority data and literature, as detailed in Table 1. Additional parameters were calibrated based on the average daily cases recorded from June 21, 2021 to July 21, 2021. To mitigate the variability of daily case numbers and enhance signal detection, we utilized the 7-day moving average (MA) of the daily cases [26,27]. Two models were constructed, each reflecting scenario 1 and scenario 2, which differed in the vaccination rate parameters employed. Scenario 1 represented a situation where the vaccination rate was designed to achieve a target of 80% of the adult population being fully vaccinated by the earliest possible date (October 31, 2021). Scenario 2 mirrored scenario 1, but the timeline to reach the target was extended to the latest possible date (December 31, 2021). In this study, the term “fully vaccinated” refers to individuals who have received both doses of the COVID-19 vaccine. We also created a model for scenario 3, which projected the trajectory of COVID-19 cases in the absence of vaccination effects. The developed SEIRV models were validated by comparing the model’s forecast with the observed case trends from July 22, 2021 to December 31, 2021.

The SEIRV model accounted for the effects of vaccination using the following parameters.

#### Weighted average vaccine efficacy

Vaccine efficacy was determined by computing the weighted average efficacy (*μ*) of 3 different vaccines, taking into account their respective distribution proportions. For 2021, the planned distribution proportions for the Pfizer, Sinovac, and AstraZeneca vaccines were reported to be 70.44%, 19.50%, and 10.06%, respectively [14]. The Pfizer and AstraZeneca vaccines demonstrated a range of efficacy against the Delta variant, with reported efficacy levels of 79-88% and 60-67%, respectively [22,23]. During the study period, there was no available information regarding the efficacy of the Sinovac vaccine against the Delta variant. Therefore, we used an efficacy level of 51% for the Sinovac vaccine, as documented by the WHO [24]. In our calculations, we used the lower bound efficacy levels for each vaccine to provide conservative estimates. These were 79%, 51%, and 60% for Pfizer, Sinovac, and AstraZeneca, respectively. Taking into account the distribution proportions of each vaccine and their efficacy levels, we estimated the weighted average vaccine efficacy for the Malaysian population to be 71.64%.

#### Daily adult population being fully vaccinated

The daily number of adults achieving full vaccination status (y) was calculated by applying a best-fit line to the observed trend of second-dose vaccinations from June 21, 2021 to July 21, 2021. This line served as the initial reference for determining the parameters that represent the vaccination rate in both scenario 1 and scenario 2.

As of July 21, 2021, 21.64% (n=5,066,963) of the adult population had been fully vaccinated [20]. This leaves a remaining 58.36% (n=13,660,717) of the adult population that needs to be vaccinated in order to reach the goal of having 80% of adults fully vaccinated, as detailed in Supplementary Material 1 [20]. The initial trend of the Phase 3 NIP vaccination was represented by the best-fit line equation y=αt^2^+ƙt+31,667. In this equation, y stands for the number of adults who have received the second dose of the vaccine, and t represents the sequence of days. The multiplication factors (α and ƙ) were adjusted to ensure that the projected lines meet the specified target. In scenario 1, the values of α and ƙ were set at 5 and 790 respectively, as outlined in Supplementary Material 2. For scenario 2, the values of α and ƙ were adjusted to 2 and, 202 respectively, also detailed in Supplementary Material 2.

### Susceptible-exposed-infectious-recovered-vaccinated mathematical model formulation

**Figure 1.**
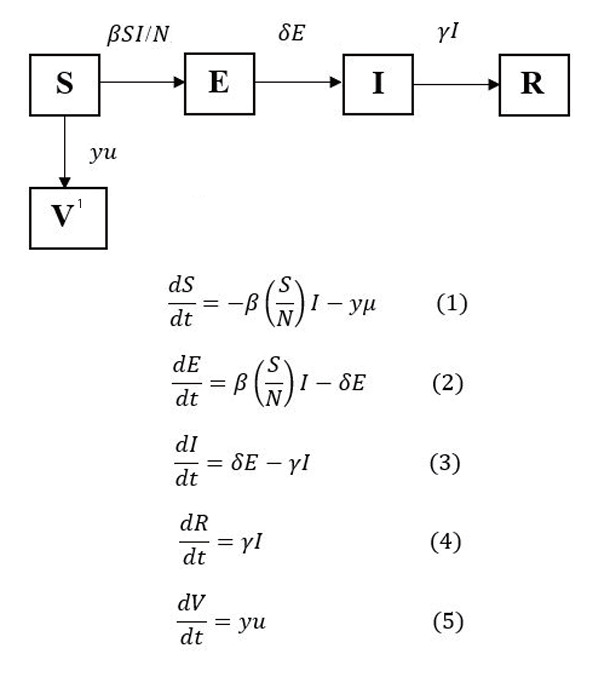
The susceptible-exposed-infectious-recovered-vaccinated model and its differential equations. ^1^For scenario 3, the *V* compartment was omitted.

The SEIRV model developed in this study has 5 compartments: susceptible (*S*), exposed (*E*), infectious (*I*), recovered (*R*), and vaccinated (*V*). *S* stands for the susceptible subpopulation that could be exposed to the virus by contact with an infected individual who is mobile and free to interact with other susceptible persons in the initial phase. *E* represents an exposed subpopulation that has been exposed to the virus but is not yet infected. The likelihood of disease transmission, *β*, governs the extent of the transition of individuals from *S* to *E*. The exposed subpopulation in *E* that subsequently becomes infectious would enter *I* after the incubation period. The duration of an individual remaining in *E* is determined by the length of the incubation period of the virus, 1/*δ*, and *δ* denotes the rate of infectiveness. In *I*, infected individuals are the source of infection in the model that can spread the virus to other susceptible individuals until they have recovered. The duration of the infectious period, 1/*γ* of the infected individuals, is determined by its rate *γ*, which is denoted as the recovery rate. The recovered individuals in *I* enter *R*. Individuals who have received 2 doses of COVID-19 vaccines are considered fully vaccinated and would be removed from *S* to enter *V*, the number of which was determined based on weighted average vaccine efficacy *μ* and the daily adult population being fully vaccinated *y*. The transition of individuals from various compartments and the differential equations (1)-(5), which describe the dynamics of COVID-19, were formulated based on the compartmental diagram described in Figure 1. The reproduction number (R_0) is the number of secondary infections caused by a primary infected individual during the infectious period ($R\_0\;=\frac{\beta }{\gamma }$)[31-33], wherein the estimated R_0 accounted for the existing PHSM effects during the study period.

The SEIRV model used in this study had the following assumptions: (1) There is a closed population with a constant size of N. As the model development and projection took place within a short period, background birth and death rates were not included in the estimation. (2) Initially, the entire Malaysian population is susceptible, hence *S*_0_=*N*. (3) There is homogeneous mixing within the population, and all individuals are assumed to have an equal likelihood of contracting and transmitting the virus. (4) Eighty percent of the adult population would be vaccinated (completed 2 doses) for scenarios 1 and 2 based on the best-fit equation of vaccine deployment from June 21, 2021 to July 21, 2021 and its projections. (5) Individuals who have completed 2 doses of vaccination are no longer susceptible to COVID-19 infection. (6) Individuals who have been infected with COVID-19 are no longer susceptible to COVID-19 reinfection.

### Ethics statement

The study was registered with the National Medical Research Register (NMRR ID-23-00165-8JR) and had obtained ethical approval from the Medical Research and Ethics Committee (reference No. 23-00165-8JR [2]).

## RESULTS

### Model calibration

**Figure 2.**
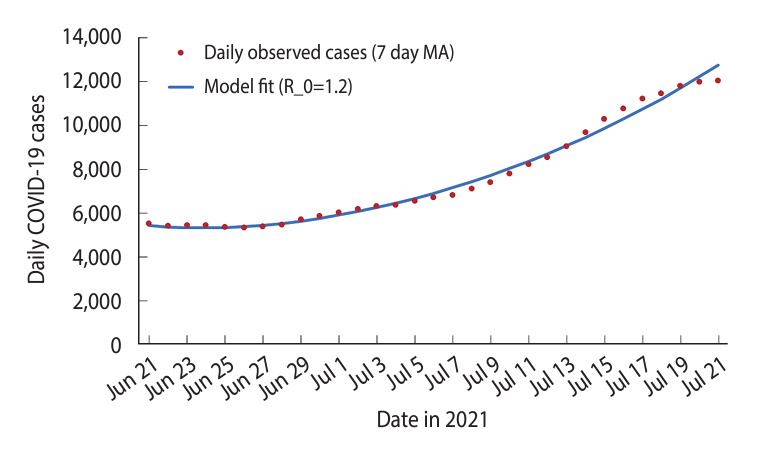
Susceptible-exposed-infectious-recovered-vaccinated model fit of daily coronavirus disease 2019 (COVID-19) cases in Malaysia, June 21 to July 21, 2021. MA, moving average.

A SEIRV model, utilizing a 7-day MA of observed COVID-19 cases, was fitted from June 21, 2021 to July 21, 2021. This model was calibrated using the least squares method [25]. The R_0 value was estimated to be 1.20, as shown in Figure 2.

### Model outputs for scenarios 1 and 2

**Figure 3.**
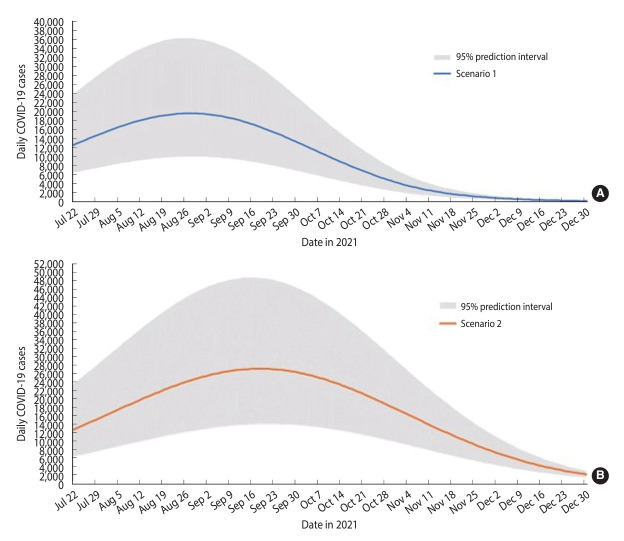
Susceptible-exposed-infectious-recovered-vaccinated model forecast of daily coronavirus disease 2019 (COVID-19) cases with 95% prediction intervals for Malaysia, July 22, 2021 to December 31, 2021. (A) Scenario 1. (B) Scenario 2.

The SEIRV model developed for scenario 1 predicted that the outbreak would peak on August 28, 2021, with a maximum of 19,614 cases reported in a single day (Table 2). Following this peak, a decline in daily cases was projected, culminating in an estimated low of 206 cases on December 31, 2021. The total number of cases forecasted for the period from July 22, 2021 to December 31, 2021—referred to henceforth as the forecast period— was 1,566,190 (Table 2 and Figure 3).

The SEIRV model developed for scenario 2 predicted that the outbreak would reach its peak on September 19, 2021, with the highest daily case count being 27,150 (Table 2). Following this peak, a decreasing trend in cases was projected until December 31, 2021, with the lowest daily case count estimated to be 2,103 on that date. The total number of forecasted cases for this period was estimated to be 2,802,980 (Table 2 and Figure 3).

Based on scenarios 1 and 2, the peak of the outbreak was projected to occur between August 28, 2021 and September 19, 2021, with an estimated 19,614 to 27,150 cases. The predicted cumulative cases during this forecast period range from 1,566,190 to 2,802,980. If we achieved full vaccination of 80% of the adult population by October 31, 2021 (scenario 1), we could prevent 1,236,790 cumulative cases. This would also result in a lower peak of 7,536 cases, compared to reaching the 80% vaccination target by December 31, 2021 (scenario 2).

### Comparison between the models of scenarios 1 and 2 with observed cases

**Figure 4.**
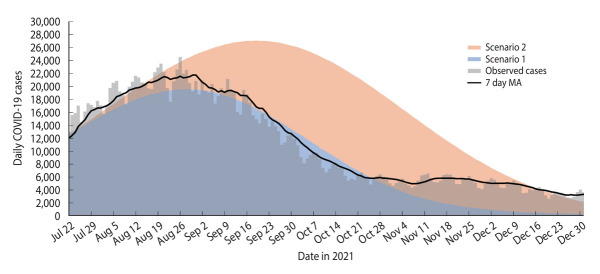
Daily observed coronavirus disease 2019 (COVID-19) cases and susceptible-exposed-infectious-recovered-vaccinated models of forecast cases for scenarios 1 and 2 in Malaysia, July 22, 2021 to December 31, 2021. MA, moving average.

The peak in observed cases occurred on August 26, 2021, 2 days earlier than predicted in scenario 1 (Figure 4). The highest daily observed case count was 24,599, which fell within the forecasted values for both scenarios 1 and 2. This count exceeded the highest daily forecasted cases in scenario 1 by 20.27% (n=4,985), but was 10.37% lower (n=2,551) than the highest daily cases forecasted in scenario 2.

The total number of observed cases reached 1,806,202, falling within the projected range for scenarios 1 and 2. This figure was 13.29% (n=240,012) higher than the predicted total cases in scenario 1, yet 55.19% (n=996,778) lower than those forecasted for scenario 2 (Table 2 and Figure 4).

The observed daily case trend closely mirrored the trend of scenario 1 from the beginning of the forecast period on July 22, 2021 until October 25, 2021. However, from October 25, 2021 to December 31, 2021, the observed case trend exceeded the forecast for scenario 1, yet remained below the forecast for scenario 2 (Figure 4).

### Model output and observed cases for scenario 3 (without vaccination)

**Figure 5.**
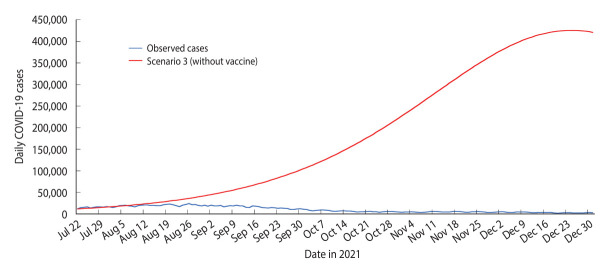
Daily observed coronavirus disease 2019 (COVID-19) cases and susceptible-exposed-infectious-recovered-vaccinated model of forecast cases for scenario 3 in Malaysia, July 22, 2021 to December 31, 2021.

The SEIRV model developed for scenario 3 predicted that the peak of the outbreak would occur on December 24, 2021, with the highest daily case count reaching 425,400 (Table 2 and Figure 5). The model also projected a cumulative of 29,762,688 cases for the forecast period (Table 2 and Figure 5).

The highest daily forecasted case count in scenario 3 (n=425,400) exceeded the observed highest daily case count (n=24,599) by 1,629.34%. Similarly, the cumulative forecasted cases in scenario 3 (n = 29,762,688) surpassed the cumulative observed cases (n=1,806,202) by 1,547.81% (Table 2, Figures 4 and 5).

## DISCUSSION

The COVID-19 pandemic was an unprecedented global health crisis and remains a global health threat. Nonetheless, the lessons learned will be valuable if there is an emergence of a novel virus in the future [1]. While PHSMs have been effective in controlling the spread of COVID-19, it is important to consider the negative socioeconomic consequences brought about by the prolonged implementation of PHSMs. COVID-19 vaccination has emerged as a promising strategy to accelerate the transition of the COVID-19 pandemic into the endemic phase [34,35]. Additionally, the large-scale introduction of multiple types of vaccines was first observed during the COVID-19 pandemic. Hence, it is crucial to study the effects of vaccination on the containment and control of COVID-19 to help guide effective vaccination strategies and public health interventions.

Compartmental models serve as effective tools for determining the impacts of vaccination, thanks to their flexible frameworks that allow for the inclusion of state variables representing vaccination effects within the model structure [36]. In our study, a strategy for accurately modeling the effects of vaccination involved considering the weighted average vaccine efficacy. This was estimated by taking into account the proportion of deployment for 3 vaccines and the predominant COVID-19 variant circulating during the study period. To generate more conservative estimates and thereby avoid underestimating projected COVID-19 cases, we used the lowest available efficacy values for the Pfizer, AstraZeneca, and Sinovac vaccines against the Delta variant when estimating the weighted average vaccine efficacy [18,19,22-24]. Furthermore, the disease transmissibility estimate (R_0= 1.20) was calibrated during the model fitting period to reflect the most recent COVID-19 transmission dynamics. Since R_0 was held constant throughout the model development, any changes in COVID-19 case trends observed during the forecast period could be attributed to the effects of vaccination.

In an effort to expedite control of the COVID-19 outbreak in Malaysia, the vaccination campaign was accelerated through the establishment of vaccination centers across the country [37]. Additionally, to address the urgent need to manage the surge of COVID-19 cases, particularly amidst the rapid transmission of the Delta VOC in Klang Valley, the most densely populated region in Malaysia, Operation Surge Capacity was implemented on July 25, 2021. This operation further increased the daily vaccination rate [38,39]. Through these combined efforts, Malaysia successfully met its goal of vaccinating the adult population by October 31, 2021. As a result, 87.36% of adults had received 2 doses since the start of Phase 3 of the NIP [20]. This accomplishment offers a plausible explanation for the close alignment of observed cases with the model forecast in scenario 1. Scenario 1, the best-case scenario in our study, was characterized by an accelerated vaccination rate. This led to an earlier, but lower peak of the outbreak compared to scenario 2. Moreover, the accelerated vaccination rate was projected to reduce the magnitude of the outbreak, potentially averting approximately 1,236,790 cumulative cases compared to scenario 2, where the vaccination rate was slower. Similar findings have been reported in studies conducted in the United States and Italy, which found that a rapid rollout of vaccinations was beneficial in controlling and managing the COVID-19 pandemic [8,40].

Among the strengths of our study, the first was the application of innovative modeling strategies. These strategies enabled us to account for several factors: (1) the impact of multiple vaccines with varying efficacy levels, (2) the differing proportions of vaccine deployment during the NIP phase, and (3) the earliest and latest scenarios for achieving an 80% fully vaccinated adult population. Our second strength was our focus on developing a straightforward model that accounted for the effects of vaccination on the susceptible population. This approach reduced model complexity, allowing us to capture the large-scale effects of vaccination. Lastly, the models we generated were capable of accurately reflecting the effects of vaccination on the outbreak trends and trajectory of COVID-19 cases. This has proven instrumental in the effective management of the COVID-19 pandemic in Malaysia.

As with all models, ours have limitations that should be considered within the context of the model’s assumptions. The models we developed in this study did not account for the diminishing effects of vaccine efficacy against COVID-19 infections. However, given that our study provided a forecast spanning 5 months, it’s worth noting that literature suggests the waning effects of vaccine efficacy typically become apparent beyond this period [41]. Furthermore, our models assume that individuals who have contracted COVID-19 are no longer susceptible to the virus. At the time these models were created, there was limited data available on breakthrough COVID-19 infections. Finally, our models did not account for the emergence of the new Omicron VOC. However, the detection of the Omicron VOC in Malaysia happened towards the end of our model’s forecast period in December 2021 [42,43].

In conclusion, our study demonstrated the effectiveness of COVID-19 vaccination in reducing COVID-19 case trends in Malaysia, even in the presence of the highly transmissible Delta VOC. The compartmental models developed successfully captured the effects of large-scale vaccination efforts and provided insight into the potential outcomes of different vaccination strategies.

## Figures and Tables

**Figure f6-epih-45-e2023093:**
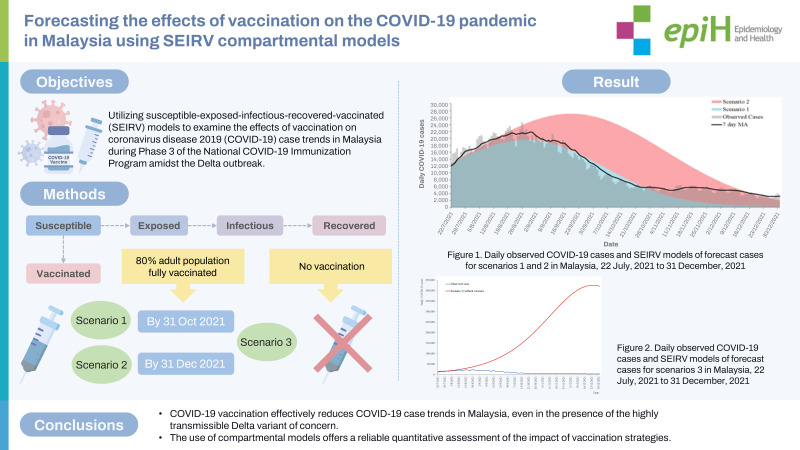


**Table 1.** Parameters and their respective values in SEIRV model development

Parameter	Description	Value	Source
N	Total population of Malaysia, 2020	32,400,000	[28]
1/*δ*	Incubation period	5.20 days	[29]
1/*γ*	Infectious period	3.95 days	[30]
*β*	Likelihood of disease transmission		Calibrated
R_0	Reproduction number		Calibrated
*y*	Daily adult population fully vaccinated		Supplementary Materials 1 and 2
*μ*	Weighted average vaccine efficacy	71.64%	[14,22-24]

SEIRV, susceptible-exposed-infectious-recovered-vaccinated.

**Table 2.** Comparison of forecasted coronavirus disease 2019 cases based on scenarios 1, 2, and 3 with observed cases in Malaysia, July 22, 2021 to December 31, 2021

Scenarios	Cumulative forecasted cases (a)	Cumulative observed cases (b)	Highest no. of daily forecasted cases (c)	Highest no. of daily observed cases (d)	Difference between forecasted and observed cumulative cases (a-b) (%)^1^	Difference between highest no. of daily cases in forecasted and observed cases (c-d) (%)^1^
Scenario 1	1,566,190	-	19,614	-	-240,012 (-13.29)	-4,985 (-20.27)
Scenario 2	2,802,980	-	27,150	-	996,778 (55.19)	2,551 (10.37)
Scenario 3	29,762,688	-	425,400	-	27,956,486 (1,547.81)	400,801 (1,629.34)
Observed cases	-	1,806,202	-	24,599	-	-

1 Observed cases were used as the denominator in estimating the percentage differences between observed cases and forecasted cases.

## SUPPLEMENTARY MATERIALS

Supplement Material 1. Cumulative adult population fully vaccinated based on Scenario 1 and 2, Malaysia, 22 July 2021 to 31 December 2021

Supplement Material 2. Daily adult population being fully vaccinated for Scenario 1 and 2, Malaysia, 22 July 2021 to 31 December 2021
