# Supplementary material for: Forecasting the effects of vaccination on the COVID-19 pandemic in Malaysia using SEIRV compartmental models
Source: Epidemiol Health. 2023 Oct 17;45:e2023093. doi: 10.4178/epih.e2023093 (PMC10867513; doi:10.4178/epih.e2023093)
Supplement: Supplement Material 1. — Cumulative adult population fully vaccinated based on Scenario 1 and 2, Malaysia, 22 July 2021 to 31 December 2021 [file epih-45-e2023093-Supplementary-1.docx]

**Supplementary Material 1**

**Supplementary Material 1.** Cumulative adult population fully vaccinated based on Scenario 1 and 2, Malaysia, 22 July 2021 to 31 December 2021
